# Whole-Genome-Guided Functional Characterization of *Limosilactobacillus fermentum* SHY0006 Reveals Hypolipidemic Activity and Improvement in Insulin Resistance  

**DOI:** 10.3390/foods15091508

**Published:** 2026-04-27

**Authors:** Zhengyang Xu, Zihan Sun, Feiyang Wang, Qingyang Han, Shuyu Li, Chunxu Xue, Yanhui Li, Dong Liu, Jun Cai, Haiyan Sun

**Affiliations:** 1 School of Life and Health Sciences, Hubei University of Technology, Wuhan 430068, China; 13273716480@163.com (Z.X.); lishuyu@mail.szpu.edu.cn (S.L.); 2 School of Food and Drug, Shenzhen Polytechnic University, Shenzhen 518000, China; szhxxn@163.com (Z.S.); wangfeiyang@szpu.edu.cn (F.W.); qy_han@outlook.com (Q.H.); xuechunxu@outlook.com (C.X.); liyanhui1@szpu.edu.cn (Y.L.); liudongsz@szpu.edu.cn (D.L.)

**Keywords:** *Limosilactobacillus fermentum*, probiotic, whole-genome sequencing, hypolipidemic potential, glycemic regulation, functional food

## Abstract

*Limosilactobacillus fermentum* SHY0006 was isolated from Miao sour soup, a traditional fermented food from Guizhou, China, and systematically evaluated for its safety, metabolic functionality, and stress adaptability using phenotypic assays combined with whole-genome sequencing. SHY0006 exhibited no hemolytic activity and harbored no detectable virulence-associated or acquired antibiotic resistance genes, supporting its safety profile. Functionally, SHY0006 improved lipid metabolism and insulin resistance in both cell and animal models. In hyperlipidemic mice, hepatic triglyceride accumulation was markedly reduced, accompanied by favorable modulation of serum lipid parameters, including LDL-C, HDL-C, and free fatty acids. In diabetic mice, the strain improved insulin tolerance test (ITT) performance, indicating enhanced systemic insulin sensitivity. Whole-genome analysis revealed complete biosynthetic pathways for riboflavin and folate, along with extensive carbohydrate utilization capacity, highlighting its metabolic versatility. In addition, SHY0006 exhibited strong tolerance to environmental stress, supporting its potential viability in food matrices and gastrointestinal conditions. Collectively, these findings suggest that SHY0006 is a safe and metabolically versatile probiotic candidate with potential applications in functional foods targeting metabolic health.

## 1. Introduction

With the increasing incidence of obesity, hyperlipidemia, and type 2 diabetes globally, traditional drug therapies, while effectively controlling relevant indicators, also bring about non-negligible side effects such as gastrointestinal reactions, hepatotoxicity, and hypoglycemia, which restricts their long-term application [1]. Consequently, the demand for safe and effective probiotics to regulate the metabolic health of the host has attracted increasing attention. Lactic acid bacteria (LAB) are among the most extensively studied probiotics due to their long history of safe use, desirable technological properties, and well-documented roles in gut microecology, immune modulation, and metabolic regulation [2,3]. Within this group, *Limosilactobacillus fermentum* has been frequently isolated from fermented foods and the human gastrointestinal tract, with several strains demonstrating antioxidant, lipid-lowering, and glucose-modulating activities [4,5,6]. However, probiotic traits are highly strain-specific, and many naturally occurring *L. fermentum* strains remain insufficiently characterized.

Traditional Guizhou Miao sour soup represents a unique fermentation ecosystem dominated by LAB, shaped by long-term spontaneous fermentation and rich microbial diversity [7]. Recent studies have revealed its beneficial effects on gut microbiota and metabolic balance, such as alleviating colitis and improving lipid metabolism in animal models [8,9]. These ecological conditions favor the emergence of LAB strains with exceptional stress tolerance and metabolic versatility, providing valuable sources for probiotic discovery.

Despite increasing interest in food-derived probiotics, few studies have integrated genomic characterization with systematic phenotypic validation to clarify the molecular basis linking probiotic genotypes to metabolic functions. The traditional methods of screening based on the physical appearance of the organism, although effective, are now unable to meet the increasing demands for high-throughput and precise analysis of the safety, functional mechanisms, and personalized applications of the strains. For example, conventional phenotypic screening cannot directly identify functional genes, virulence-associated determinants, or antibiotic resistance loci, all of which are increasingly important in probiotic evaluation [10]. Whole-genome sequencing (WGS) has emerged as a powerful tool for elucidating the genetic foundations of probiotic functionality, enabling precise identification of genes related to safety, metabolic versatility, and stress response [11,12,13]. However, comprehensive studies that combine genomic annotation with experimental verification remain limited, particularly in evaluating how these genetic traits translate into physiological resilience and host metabolic regulation. Integrating WGS-based insights with in vitro and in vivo functional assays can therefore provide a more mechanistic understanding of probiotic efficacy and support strain-specific probiotic development [14].

In this study, a strain of *Limosilactobacillus fermentum*, designated SHY0006, was isolated from Miao sour soup, a traditional fermented food from Guizhou, China. Its safety, stress tolerance, gastrointestinal survivability, aggregation capacity, antioxidant activity, and metabolic regulatory functions were systematically evaluated through a comprehensive set of in vitro, in vivo, and genomic analyses. These assessments aim to provide a scientific foundation for the potential application of SHY0006 as a novel probiotic candidate for improving glucose and lipid metabolism and for developing functional foods targeting metabolic disorder.

## 2. Materials and Methods

### 2.1. Strain Isolation and Identification

Samples of Miao sour soup were collected aseptically from Guizhou, China, and serially diluted in sterile water. Aliquots (100 µL) were spread on de Man–Rogosa–Sharpe (MRS) agar (Guangdong Huankai Microbial Sci. & Tech. Co., Ltd., Guangzhou, China) and incubated anaerobically at 37 °C for 48 h. A single colony with typical *Lactobacillus* morphology was purified and identified by 16S rRNA sequencing using universal primers 27F/1492R. The sequence was queried against the NCBI 16S database (https://blast.ncbi.nlm.nih.gov/Blast.cgi; accessed on 20 October 2024) via BLASTn, and phylogenetic assignment was confirmed by average nucleotide identity (ANI) analysis with the type strain *L. fermentum* ATCC 14931.

### 2.2. Safety Assessment

#### 2.2.1. Antibiotic Susceptibility

Antibiotic susceptibility was evaluated using the Kirby–Bauer disk diffusion method. A 0.1 mL aliquot of *Limosilactobacillus fermentum* SHY0006 suspension (~1.0 × 10^9^ CFU/mL) was evenly spread on sterilized MRS agar plates. Antibiotic disks (Hangzhou Microbial Reagent Co., Ltd., Hangzhou, China) were placed onto the agar surface using sterile forceps and left at room temperature for 40 min, followed by incubation at 37 °C for 24 h. Each antibiotic was tested in triplicate. Susceptibility interpretations were performed according to previously reported inhibition zone criteria for lactic acid bacteria based on Clinical and Laboratory Standards Institute (CLSI) guidelines for Gram-positive bacteria (CLSI M100, 35th edition, 2025), together with European Food Safety Authority (EFSA) recommendations for evaluating acquired antimicrobial resistance in food-related bacteria, and were further supported by whole-genome analysis of resistance determinants [15].

#### 2.2.2. Hemolytic Activity

The strain was activated in MRS broth at 37 °C for 12 h. *Staphylococcus aureus* ATCC 12598 was used as a positive control. Both the control and SHY0006 strain were streaked onto Columbia blood agar plates (Guangdong Huankai Microbial Sci. & Tech. Co., Ltd., Guangzhou, China) and incubated at 37 °C for 24 h to assess hemolytic activity based on colony morphology and hemolysis zones.

### 2.3. Evaluation of Potent Hypolipidemic Activity in HepG2 Cells, Zebrafish, and Mouse Models

#### 2.3.1. Hypolipidemic Effects of Probiotic Fermentation Supernatant on HepG2 Cells

HepG2 cells (Sevier Biotechnology Co., Ltd., Wuhan, China) were seeded at 3 × 10^4^ cells/well and induced with 500 µM sodium oleate for 24 h. Cells were treated with 5% (*v*/*v*) CFS for 24 h. Lipid droplets were stained with BODIPY 493/503 and quantified by high-content imaging (PerkinElmer Harmony). Lipid droplet area fraction (FDA) was calculated as follows:

(1) $$\mathrm{FDA}=\frac{A_{LD}}{A_{\mathrm{cyto}}}$$

where A_LD_ is the total lipid droplet area and A_cyto_ is the cytoplasmic area per cell.

#### 2.3.2. Hypolipidemic Effects in the Liver of High-Fat Diet-Induced Zebrafish

The adult zebrafish used in the experiment were of the AB strain and were obtained from the National Zebrafish Resource Center. Zebrafish embryos at 2 days post fertilization (dpf) were exposed to 0.003% phenylthiourea (PTU) to suppress melanogenesis and improve optical transparency for subsequent imaging analysis. All groups were subjected to identical PTU treatment conditions to ensure that any potential PTU-related effects were consistent across treatments. At 5 dpf, larvae were fed 0.1% egg yolk powder for 2 days to establish a hyperlipidemic model. Lipid accumulation in the liver was visualized by Oil Red O (ORO) staining.

Four groups were prepared: control, model, positive control (fenofibrate, 1.5 mg/L), and probiotic groups. All groups except the control were treated with E3 medium (5 mM NaCl, 0.17 mM KCl, 0.33 mM CaCl_2_, and 0.33 mM MgSO_4_) supplemented with 0.1% (*w*/*v*) egg yolk. The probiotic group received bacterial suspension at 1 × 10^7^ CFU/mL and was incubated at 28.5 ± 0.5 °C for 48 h, with medium refreshed every 24 h.

After treatment, zebrafish were fixed in 4% paraformaldehyde overnight and stained with ORO for 2 h. Liver lipid deposition was observed using a stereomicroscope, and the fluorescence intensity of the liver area was quantified by using ImageJ software (v1.53c). The standardized effect size (Cohen’s d) was calculated to evaluate the lipid-lowering effect:

(2) $$\text{Cohen’s }d=\frac{{\mathrm{Mean}}_{M}-{\mathrm{Mean}}_{T}}{S_{p}}$$

Mean_M_: the average liver brightness of the model group; Mean_T_: the average liver brightness of the experimental group;

#### 2.3.3. Hypolipidemic Effects on Lipid Metabolism in High-Fat Diet (HFD) Mice

Thirty male C57BL/6 mice with high-fat diet-induced obesity (after 8 weeks of high-fat feeding) and fifteen age-matched male C57BL/6 mice fed a normal diet (13 weeks old, specific pathogen-free) were purchased from Guangdong Yaokang Biotechnology Co., Ltd. (Guangzhou, China). (animal use license: SYXK (Su) 2023-0036; production license: SCXK (Su) 2023-0009). The nutritional compositions of the high-fat diet and control diet are shown in Table 1.

Before the experiment, the micro-barrier housing system and cages were disinfected and sterilized. Mice were maintained under specific pathogen-free conditions at 25 ± 2 °C, relative humidity of 50 ± 10%, with a 12 h light/dark cycle, and were allowed ad libitum access to food and sterile water. Bedding and sterile water were replaced every three days. After one week of acclimatization, the 30 high-fat diet-induced obese mice were randomly assigned to three experimental groups (n = 10 per group) using a computer-generated randomization list, while 15 mice fed a normal diet under the same conditions served as the normal control group (NCD). The sample size (n = 10 per group) was determined based on previous high-fat diet and probiotic intervention studies, which, assuming an effect size reflected by differences in body weight gain, provides >80% statistical power at α = 0.05.

Mice in the NCD group received sterile saline (0.15 mL/10 g body weight per day) by oral gavage and were fed the control diet. Mice in the high-fat diet group (HFD) were fed the high-fat diet without treatment. The atorvastatin group received atorvastatin solution (0.17 mg/mL, 0.15 mL/10 g body weight per day; equivalent to 2.6 mg/kg body weight) by oral gavage while being fed the high-fat diet. The probiotic treatment group received a suspension of *Limosilactobacillus fermentum* SHY0006 at a concentration of 1 × 10^9^ CFU/mL (0.15 mL/10 g body weight per day) by oral gavage together with the high-fat diet. All treatments were administered once daily for 30 consecutive days.

During the experimental period, body weight and food intake were recorded daily to monitor the growth status of the mice, and the first day of probiotic administration was defined as day 0 of the intervention experiment. At the end of the experiment, mice were fasted overnight for 12 h, and fasting body weight was recorded. Blood samples were collected from the orbital sinus, allowed to stand at room temperature, and centrifuged at 2000× *g* for 10 min to obtain serum, which was stored at −80 °C for further biochemical analysis. After blood collection, mice were sacrificed, and liver and epididymal adipose tissues were excised and weighed. Tissues were divided into two portions: one portion was fixed in 4% paraformaldehyde, and the other was snap-frozen in liquid nitrogen and stored at −80 °C for subsequent analyses. Hepatic total cholesterol (TC) and triglyceride (TG) levels, as well as serum levels of high-density lipoprotein cholesterol (HDL-C), low-density lipoprotein cholesterol (LDL-C), and free fatty acids (FFAs), were measured using commercial assay kits (Solarbio Biotechnology Co., Ltd., Beijing, China) according to the manufacturers’ instructions. All animal procedures were approved by the Animal Ethics Committee of Shenzhen Polytechnic University and conducted in accordance with relevant guidelines for laboratory animal care and use.

### 2.4. Evaluation of Potent Hypoglycemic Activity in C2C12 Cells, Zebrafish, and Mouse Models

#### 2.4.1. Hypoglycemic Effects in Insulin-Resistant C2C12 Cells

Insulin resistance (IR) in C2C12 (Sevier Biotechnology Co., Ltd., Wuhan, China) myotubes was induced using 100 nM insulin for 72 h. Cells were treated with 5% (*v*/*v*) probiotic supernatant during the last 24 h. Glucose uptake was measured using 50 µM 2-NBDG (1 h) and flow cytometry.

#### 2.4.2. Hypoglycemic Effects in Diabetic Zebrafish

All animal experiments were conducted in accordance with approved ethical guidelines (see Ethical Approval section).

Zebrafish (AB strain) embryos were obtained from the National Zebrafish Resource Center (China). A 4% glucose solution was prepared in E3 medium to induce hyperglycemia. Larvae at 3 days post-fertilization (dpf) were exposed to the glucose solution for 48 h, with the medium refreshed every 24 h.

Treatment groups included normal control (NC), model (DM), positive control (acarbose, 1.6 mg/mL), and probiotic groups (1 × 10^6^ CFU/mL). Each group contained 40 larvae per well in 6-well plates, with three replicates. All groups except the control were maintained in 4% glucose E3 medium at 28.5 ± 0.5 °C for 48 h, with medium renewal once daily.

After exposure, larvae were washed with E3 medium, centrifuged gently to remove excess solution, and homogenized at 4 °C with zirconium beads. After centrifugation (13,000 rpm, 5 min, 4 °C), glucose content in the supernatant was determined using a glucometer.

#### 2.4.3. Hypoglycemic Effects in Diabetic Mice (GTT, ITT, FBG)

Animal source, housing conditions, randomization procedures, sample size determination, and ethical approval were identical to those described in Section 2.3.3, except that obesity was induced by 12 weeks of high-fat feeding before intervention.

Mice in the NCD group received sterile saline (0.15 mL/10 g body weight per day) by oral gavage and were fed the control diet. Mice in the HFD group were fed the high-fat diet without treatment. The pioglitazone group received pioglitazone solution (0.67 mg/mL, 0.15 mL/10 g body weight per day) by oral gavage (Takeda Pharmaceutical Company Limited, Osaka, Japan), while the probiotic treatment group received *Limosilactobacillus fermentum* SHY0006 suspension (1 × 10^9^ CFU/mL, 0.15 mL/10 g body weight per day). All treatments were administered once daily for 30 consecutive days.

A glucose tolerance test (GTT) was performed on day 22. After fasting for 10 h, mice were intraperitoneally injected with glucose solution (2 g/kg body weight). Blood samples were collected from the tail vein at 0, 30, 60, 90, and 120 min, and blood glucose levels were measured using a glucometer (LifeScan, Inc., Milpitas, CA, USA). The area under the curve (AUC) was calculated to evaluate glucose tolerance.

An insulin tolerance test (ITT) was conducted on day 27. After fasting for 6 h, mice were intraperitoneally injected with insulin (1 IU/kg body weight). Blood glucose levels were measured at 0, 30, 60, 90, and 120 min after insulin injection, and the AUC was calculated.

At the end of the experiment, mice were fasted overnight for 12 h, and fasting body weight was recorded. Blood samples were collected from the orbital sinus, allowed to stand at room temperature, and centrifuged at 2000× *g* for 10 min to obtain serum, which was stored at −80 °C for further biochemical analysis. Fasting blood glucose (FBG) levels were determined using a commercial glucose assay kit (Solarbio Biotechnology Co., Ltd., Beijing, China) according to the manufacturer’s instructions.

### 2.5. Whole-Genome Sequencing

Genomic DNA (1 μg) was prepared following the TruSeq DNA protocol (Illumina, 15026486 Rev.C). Libraries were quantified with Qubit 3.0, assessed on an Agilent 2100 Bioanalyzer, and normalized (≥10 nM) by qPCR (Bio-RAD CFX96, iQ SYBR Green kit). Pooled libraries were sequenced on a PromethION R10.4.1 flow cell (Oxford Nanopore Technologies, Oxford, UK) for 48–72 h. Short reads were quality-filtered with Trimmomatic (v0.38) and summarized with MultiQC (v1.31). Hybrid assembly was performed using Unicycler (v0.5.1) with both Illumina and Nanopore reads, and genome quality was evaluated with CheckM (v1.2.4).

### 2.6. Genomic Component Analysis

Prokka (v1.14.5) software was used to predict the coding region in the genome assembly results. The non-coding RNA (ncRNA) was predicted by RNAmer (v1.2), tRNAscan-SE (v1.3.1), and Rfam (v9.1). Prophage was predicted with PhiSpy (v3.7.8). GTDB-TK and Fasttree (v2.0.0) were used to construct a phylogenetic tree.

### 2.7. Genomic Safety Analysis

Antibiotic resistance genes (ARGs) were identified using RGI (v6.0.0) with CARD (v4.0.0), ResFinder (v4.0), and AMRFinderPlus (v3.10.42). Virulence factors (VFs) were predicted by BLASTN searches against VFDB (setB) and verified with VirulenceFinder (v2.0.3). Pathogenicity-related genes (PGs) were detected via BLASTP against PHI-base (v4.14). Mobile genetic elements (MGEs) were annotated with PlasmidFinder (v2.0.1) for plasmids and Phigaro (v2.3.0) for prophages. Predictions were filtered at ≥80% identity and ≥70% coverage for high confidence.

### 2.8. Genomic Functional Annotation Analysis

The protein sequences of predicted genes were compared with Gene Ontology (GO), Kyoto Encyclopedia of Genes and Genomes (KEGG), Cluster of Orthologous Groups of proteins (COG), Carbohydrate-Active enZymes Database (CAZy) using EggNOG-mapper (v2) software.

### 2.9. Antioxidant Activity

DPPH radical scavenging capacity was assessed using a 0.2 mmol/L DPPH ethanol solution prepared from 5 mM stock and stored in the dark. Cell suspensions (10^5^–10^8^ CFU/mL) and cell-free supernatants (CFS) were incubated with DPPH at 30 °C for 30 min in the dark, then centrifuged (8000 rpm, 10 min). Absorbance of the supernatant was measured at 517 nm, and scavenging activity (%) was calculated as follows:

(3) $$\begin{array}{l} \mathrm{DPPH}\;\mathrm{scavenging}\;\mathrm{rate}\;(\%)=[1-(A1-A2)/A3]\;\times \;100\% \end{array}$$

A1—with DPPH, sample, and ethanol; A2—no DPPH, sample present, and ethanol present; A3—DPPH present, no sample, and ethanol present

### 2.10. Environmental Stress Tolerance

#### 2.10.1. Tolerance to Different Temperatures

The growth of bacteria at different temperatures was explored by inoculating 100 μL above bacterial resuspension in 5 mL MRS broth at 4, 25, 30, 35, 37 and 45 °C for 12 h, respectively. The growth of the bacteria was evaluated by using a cell counter (Guangzhou BodBoge Technology Co., Ltd., Guangzhou, China) to determine the total number of bacterial cells.

#### 2.10.2. Tolerance to Different pH Values

To assess bacterial tolerance under different pH conditions, 100 μL of bacterial resuspension was added to 5% physiological saline adjusted to pH 2.0, 3.0, 4.0, 5.0, 6.0, and 7.0 (control), respectively. After incubation for 4 h at 37 °C, viable bacterial counts were determined using a microbial cell counter.

#### 2.10.3. Tolerance to Different NaCl Concentrations

Overnight cultures were centrifuged (8000 rpm, 10 min, 4 °C), washed twice with PBS, and resuspended to 1.0 × 109 CFU/mL. A 100 μL aliquot was inoculated into 10 mL MRS broth containing 0–10% NaCl (*w*/*v*). After 24 h incubation at 37 °C, viable cell counts were determined using a microbial cell counter.

#### 2.10.4. Tolerance to Simulated Gastric and Intestinal Fluids

Simulated gastric fluid (SGF) was formulated from 3 mg of pepsin (Shanghai Sangong Bioengineering Technology Service Co., Ltd., Shanghai, China) dissolved in 1 mL of 0.5% saline (*w*/*v*) at pH 2.0, 3.0 and 4.0.

Simulated intestinal fluid (SIF) included primary simulated intestinal fluid (PSIF) and secondary simulated intestinal fluid (SSIF). PSIF was prepared from 1 mg/mL trypsin solution (Shanghai Yuanye Biotechnology Co., Ltd., Shanghai, China) with pH 8.0, and SSIF was prepared by adding 0.03 g bile salt (Solarbio Biotechnology Co., Ltd., Beijing, China) to 10 mL PSIF.

The above solutions were filtered through a 0.22 μm filter to remove microorganisms. The bacterial resuspensions were added to SGF or SIF, respectively, and mixed for 60 s. The mixtures were then incubated at 37 °C and samples were collected separately every hour. The number of viable bacteria was measured using a microbial cell counter, and the survival rate (%) was calculated using the following equation:

(4) Survival rate (%)=[CFU (NFinal)/(CFU (NInitial)] × 100%

#### 2.10.5. Tolerance to Different Bile Salt Concentrations

To assess the tolerance of bacteria to different concentrations of bile salts, the 100 μL resuspended bacteria were inoculated into 10 mL MRS broth containing bile salt at concentrations of 0.1, 0.2, 0.3 and 0.5% (*w*/*v*). After incubation for 4 h at 37 °C, the number of viable bacteria was measured using a microbial cell counter and survival (%) was calculated using Equation (4).

#### 2.10.6. Self-Aggregation and Co-Aggregation Ability

According to the method described in [16], with slight modifications, the self-aggregation capacity was tested by placing a resuspension of *L. fermentum* SHY0006 at 25 °C for 24 h. *E. coil* 200109, *S. aureus* ATCC 12598 were selected as co-aggregation objects. The co-aggregation ability was studied by mixing the resuspension of *L. fermentum* SHY0006 and two indicator bacteria in a ratio of 1:1 (*v*/*v*) and standing at 25 °C for 24 h. The absorbance value of the upper suspension was measured at OD_600_ every 2 h.

The self- and co-aggregation rates were calculated from the absorbance value and the following equation:

(5) $$\mathrm{Aggregation}\;\mathrm{rate}\;(\%)=(1-\mathrm{Real}-\mathrm{time}\;{\mathrm{OD}}_{600}/\mathrm{Initial}\;{\mathrm{OD}}_{600})\;\times \;100\%$$

### 2.11. Statistical Analysis

The sample size (n = 10 per group) was determined based on previous studies on high-fat diet–induced obesity and probiotic interventions. Assuming an effect size reflected by differences in body weight gain, this sample size provides >80% statistical power at α = 0.05. For parametric analyses, data were tested for normality using the Shapiro–Wilk test and for homogeneity of variances using Levene’s test. All data are expressed as the mean ± standard deviation (SD). Statistical analyses and graphing were performed using GraphPad Prism software (version 10.1.2). Each experiment was performed with at least three independent biological replicates. For comparisons involving more than two groups, one-way analysis of variance (ANOVA) was conducted, followed by Tukey’s post hoc test for multiple comparisons. For high-dimensional datasets, *p* values were adjusted for multiple comparisons using the Benjamini–Hochberg false discovery rate (FDR) correction. A *p* value < 0.05 was considered statistically significant.

## 3. Results and Discussion

### 3.1. Strain Isolation and Genomic Characterization

**Figure 1:**
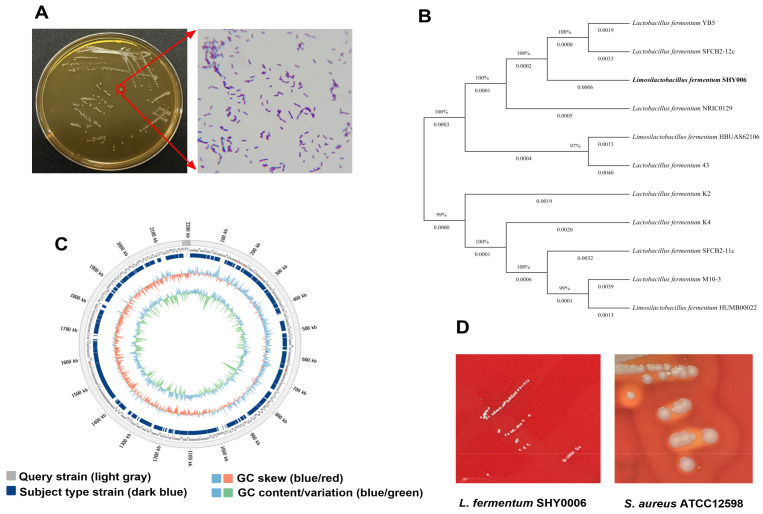
Identification of *L. fermentum* SHY0006. (**A**) Colony morphology and microscopy with Gram stain; (**B**) phylogenetic tree of *L. fermentum* SHY0006; (**C**) genome circle maps; (**D**) hemolytic activity of *L. fermentum* SHY0006 and *S. aureus* ATCC12598.

A Gram-positive bacterial strain was isolated from the traditional Miao sour soup in Guizhou region using MRS agar plates. The strain produced milky white, relatively large, circular convex colonies with moist surfaces and rough edges (Figure 1A). Based on 16S rRNA gene sequence analysis, the strain was identified as *L. fermentum* and designated as *L. fermentum* SHY0006. Average nucleotide identity (ANI) analysis further revealed 98.88% identity with the type strain *L. fermentum* ATCC 14931, confirming its taxonomic assignment. The strain has been deposited at the Guangdong Microbial Culture Preservation Centre under the accession number GDMCC 65909. To elucidate its phylogenetic position, a whole-genome-based phylogenetic tree was constructed using closely related *L. fermentum* genomes retrieved from the NCBI database (Figure 1B). The analysis revealed that SHY0006 is most closely related to *L. fermentum* strains SFCB2-12c and NRIC0129 (Figure 1B). WGS showed that SHY0006 possesses a single circular chromosome without plasmids (Figure 1C), with a total genome size of 2.20 Mb and a GC content of 51.36%. The genome encodes 2130 predicted protein-coding genes, with an average gene length of 1032 bp, and includes 58 tRNAs genes, 15 rRNAs genes, and 1 tmRNAs genes, covering all 20 standard amino acid tRNAs. The complete genome sequence of SHY0006 has been deposited in the NCBI GenBank under accession number JBSOQG000000000.

### 3.2. Safety Assessment: Absence of Antibiotic Gene, Hemolytic Activity and Virulence Factors

#### 3.2.1. Antibiotic Susceptibility

When probiotics exhibit antibiotic resistance, they may transfer this trait to pathogenic bacteria, potentially leading to significant complications [5,17]. To evaluate the safety of *L. fermentum* SHY0006, genomic and phenotypic analyses were conducted. Whole-genome analysis based on the CARD database using RGI (strict and perfect hits) revealed no acquired antibiotic resistance genes (Supplementary Table S2), indicating a low risk of resistance gene transmission. Phenotypic testing was performed against a panel of commonly used antibiotics, including β-lactams, macrolides, tetracyclines, quinolones, aminoglycosides, glycopeptides, Penicillins, and chloramphenicols (Table 2). *L. fermentum* SHY0006 exhibited resistance to a glycopeptide antibiotic (vancomycin) and an aminoglycoside antibiotic (kanamycin), showed intermediate sensitivity to cefoperazone, ceftazidime, amikacin, and ofloxacin, and was sensitive to all other tested antibiotics. The antibiotic resistance genes in the entire genome of SHY0006 were screened, and no known genes related to kanamycin and vancomycin resistance were detected. The observed resistance phenotypes may result from the inherent physiological characteristics of the bacteria: cell membrane permeability, drug efflux mechanisms, or modifications of the targets [18], rather than being mediated by horizontally transferable acquired resistance genes. Based on the analysis of its genome and phenotype, this strain supports its safety profile as a probiotic candidate.

#### 3.2.2. Absence of Hemolytic Activity and Virulence Factors

Genome annotation against the VFDB database [19] revealed no virulence factors or hemolysin-related genes in *L. fermentum* SHY0006. Consistently, the strain exhibited no hemolytic activity on blood agar plates. In contrast to the positive control, *Staphylococcus aureus* ATCC12598, which displayed a distinct hemolytic zone, SHY0006 produced opaque, non-hemolytic colonies (Figure 1D), suggesting no cytotoxic effect on red blood cells. Having confirmed the safety of SHY0006, we next explored its environmental adaptability, adhesion capacity, and metabolic potential, which together determine its probiotic functionality in the gastrointestinal tract.

### 3.3. Evaluation of Potent Hypolipidemic Activity in HepG2 Cells, Zebrafish, and Mouse Models

#### 3.3.1. Hypolipidemic Effects of Probiotic Fermentation Supernatant on HepG2 Cells

HepG2, a human hepatocellular carcinoma cell line, is characterized by a short growth cycle, stable differentiation, and unlimited proliferative capacity, and expresses key enzymes involved in lipid metabolism, including HMGCR and ATGL [20]. Using this approach, ref. [21] demonstrated that cell-free extracts of *L. acidophilus* NX2-6 reduced TG and LDL-C levels in HepG2 cells and significantly modulated the expression of lipid metabolism-related proteins, including SREBP, FAS, and CPT1.

**Figure 2:**
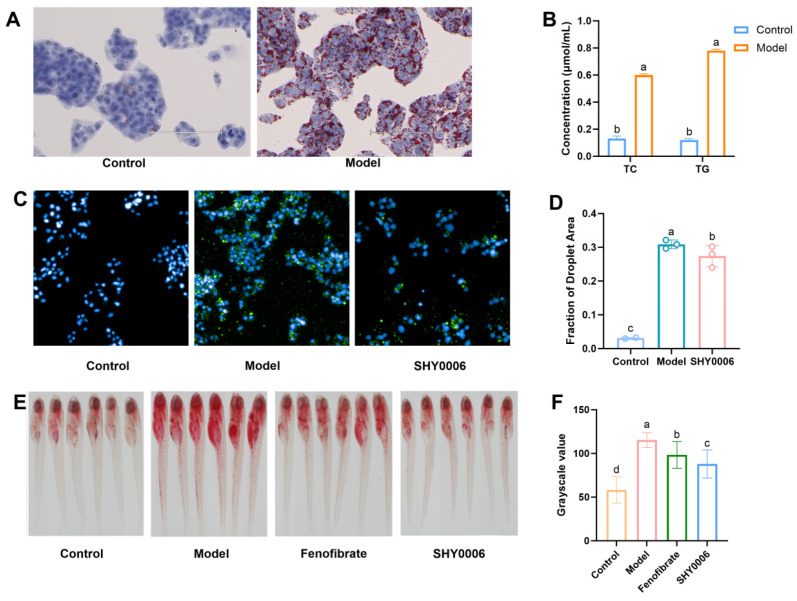
Hypolipidemic effects of *L. fermentum* SHY0006 in cellular and zebrafish models. (**A**) Representative Oil Red O staining images of lipid accumulation in sodium oleate-induced HepG2 cells. (**B**) Quantitative analysis of intracellular lipid content in HepG2 cells based on Oil Red O extraction assay (absorbance at 510 nm). (**C**) Representative BODIPY 493/503 fluorescence staining images of lipid droplets in HepG2 cells. (**D**) Quantification of lipid droplet area fraction (%) in HepG2 cells. (**E**) Representative Oil Red O staining images of hepatic lipid deposition in hyperlipidemic zebrafish larvae. (**F**) Quantitative analysis of hepatic lipid accumulation in zebrafish larvae (relative optical density). Data are presented as mean ± SD (n ≥ 3 independent experiments or biological replicates). Statistical significance was determined by one-way ANOVA followed by multiple comparison tests. Different lowercase letters indicate significant differences among groups (*p* < 0.05).

To evaluate the lipid-lowering potential of SHY0006, a high-fat HepG2 cell model was successfully established. Treatment with 0.5 mM sodium oleate significantly increased intracellular total cholesterol (TC) and triglyceride (TG) levels in the model group compared with the control group (*p* < 0.001). Oil Red O (ORO) staining further confirmed marked intracellular lipid droplet accumulation, indicating the successful establishment of a lipid-loaded HepG2 cell model (Figure 2A,B). For efficient quantitative analysis, a high-content screening method based on BODIPY 493/503 fluorescence staining was developed. The results showed that the lipid droplet area fraction in the model group (0.309 ± 0.01 μmol/mL) was significantly higher than in the control group (0.031 ± 0.001 μmol/mL) (*p* < 0.001), confirming the reliability of this method. Using this model for evaluation, the fermented supernatant of SHY0006 significantly reduced lipid droplet content in the model cells (0.274 ± 0.031 μmol/mL) (*p* < 0.05), preliminarily indicating its ability to ameliorate fat accumulation in hepatocytes (Figure 2C,D).

Previous studies have reported that metabolites derived from lactic acid bacteria, including organic acids and other fermentation products, can influence hepatic lipid metabolism by regulating key lipid metabolic enzymes and signaling pathways [22]. Therefore, the lipid-lowering effect observed in this cellular model provides preliminary evidence that SHY0006 may exert metabolic regulatory effects through microbial metabolites.

#### 3.3.2. Hypolipidemic Effects in the Liver of High-Fat Diet-Induced Zebrafish

There is about 70% homology between the zebrafish genome and the human genome [23], and the structure and functional organization of the zebrafish intestine are similar to those of mammals; moreover, its behavioral patterns and endocrine responses generally correspond well with clinical observations [24]. The lipid-lowering activity of SHY0006 was further evaluated in a zebrafish hyperlipidemia model induced by feeding 0.1% egg yolk powder. ORO staining results showed that the luminance value in the liver area of the model group (115.26 ± 8.43) was significantly higher than that of the control group (58.20 ± 14.85) (*p* < 0.001), indicating severe hepatic steatosis. The positive control drug, fenofibrate, effectively ameliorated this condition, confirming the successful establishment of the model (Figure 2E). After intervention with a bacterial suspension (1 × 10^7^ CFU/mL) for 48 h, the SHY0006 treatment group showed a significant difference in hepatic lipid deposition (87.922 ± 16.147) compared to the model group (*p* < 0.05), demonstrating the strain’s potential to improve liver lipid metabolism at the whole-animal level (Figure 2F).

The significant reduction in hepatic lipid accumulation following SHY0006 treatment suggests that the strain may influence systemic lipid homeostasis rather than exerting effects limited to isolated cells. Importantly, the consistency between the HepG2 cell results and the zebrafish model indicates that the lipid-lowering activity of SHY0006 may involve mechanisms that are conserved across biological systems. Such cross-model validation strengthens the reliability of the observed hypolipidemic effect.

#### 3.3.3. Hypolipidemic Effects on Lipid Metabolism in High-Fat Diet (HFD) Mice

Compared with the rat model, mice are cheap, easy to feed, stable blood lipids, and simple to operate in experiments, so they are also commonly used in the study of screening lipid-lowering traditional Chinese medicine and probiotics [25]. To assess the hypolipidemic efficacy of SHY0006 in a mammalian system, high-fat diet (HFD)-induced hyperlipidemic mice were orally administered SHY0006 for 30 days.

**Figure 3:**
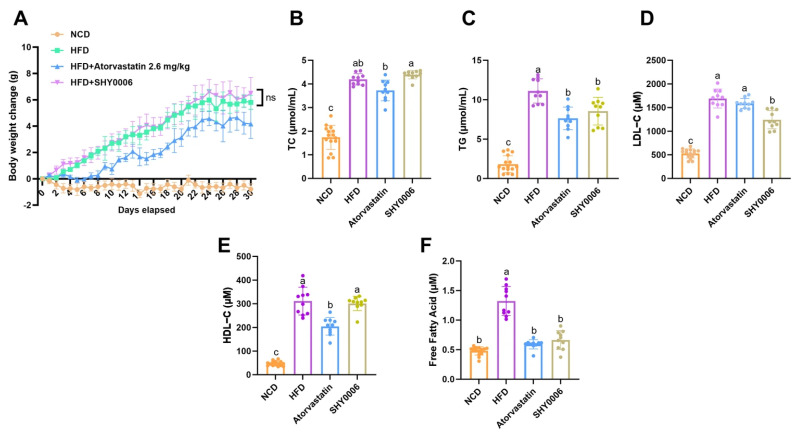
Effects of *L. fermentum* SHY0006 on lipid metabolism in HFD-induced hyperlipidemic mice. (**A**) Body weight changes in mice fed a normal chow diet (NCD), high-fat diet (HFD), HFD plus atorvastatin (2.6 mg/kg), or HFD supplemented with SHY0006 for 30 days. (**B**,**C**) Hepatic total cholesterol (TC) and triglyceride (TG) contents (μmol/mL) in different groups. (**D**) Serum low-density lipoprotein cholesterol (LDL-C, μM). (**E**) Serum high-density lipoprotein cholesterol (HDL-C, μM). (**F**) Serum free fatty acid levels (FFA, μM). Data are presented as mean ± SD (n = 6–8 per group). Different lowercase letters above the bars indicate statistically significant differences among groups (*p* < 0.05), as determined by one-way ANOVA followed by multiple comparison analysis. “ns” indicates no significant difference.

HFD feeding significantly increased body weight relative to the normal chow diet (NCD) group (*p* < 0.001), confirming the successful establishment of diet-induced obesity (Figure 3A). SHY0006 supplementation showed a modest inhibitory trend in body weight gain; however, the difference was not statistically significant (*p* > 0.05).

Hepatic lipid analysis demonstrated marked lipid accumulation in the HFD group. Total cholesterol (TC) and triglyceride (TG) levels reached 4.19 ± 0.24 and 11.11 ± 1.55, respectively, both significantly higher than those in the NCD group (1.740 ± 0.510 and 1.82 ± 1.08; *p* < 0.001) (Figure 3B,C). Atorvastatin treatment reduced TC and TG to 3.72 ± 0.44 and 7.63 ± 1.43, respectively. SHY0006 supplementation selectively decreased hepatic TG to 8.55 ± 1.76, corresponding to a 23.06% reduction compared with the HFD group, whereas its effect on TC (4.19 ± 0.24) was minimal.

Consistently, serum lipid parameters reflected systemic metabolic improvement. Serum free fatty acid (FFA) levels were markedly elevated in the HFD group (1.32 ± 0.25 nmol/μL) compared with controls (0.48 ± 0.07 nmol/μL, *p* < 0.0001) (Figure 3F). Both atorvastatin and SHY0006 significantly reduced FFA levels to 0.59 ± 0.08 and 0.66 ± 0.16 nmol/μL, respectively (*p* < 0.0001 vs. HFD group), indicating attenuation of excessive lipid mobilization. Serum LDL-C levels were also substantially increased in the HFD group (1690.92 ± 199.69 μM) relative to controls (527.60 ± 96.40 μM, *p* < 0.0001) (Figure 3D). While atorvastatin moderately decreased LDL-C to 1586.42 ± 106.42 μM, SHY0006 further reduced it to 1241.18 ± 192.26 μM (*p* < 0.0001 vs. HFD group), suggesting improved regulation of circulating low-density lipoproteins. Although HDL-C levels were elevated in the HFD group compared with controls (Figure 3E), SHY0006 treatment further increased HDL-C concentrations relative to the HFD group, implying a potential enhancement in cholesterol transport capacity.

The lipid metabolic improvements observed in the HFD mouse model further demonstrate the potential of SHY0006 to regulate host lipid homeostasis in a mammalian system. Notably, SHY0006 selectively reduced hepatic triglyceride accumulation without significantly affecting total cholesterol levels, suggesting that its metabolic effects may primarily target triglyceride metabolism and fatty acid mobilization rather than cholesterol biosynthesis [26]. The significant reductions in circulating FFA and LDL-C levels further indicate an improvement in systemic lipid metabolism. Elevated FFA levels are commonly associated with increased lipolysis and metabolic dysregulation in obesity and hyperlipidemia [27]. Therefore, the decrease in FFA levels following SHY0006 supplementation suggests a potential attenuation of excessive lipid mobilization.

Interestingly, the metabolic benefits observed in this study occurred without a significant reduction in body weight, implying that the lipid-regulating effects of SHY0006 may occur independently of weight loss. Similar observations have been reported for several probiotic strains, where metabolic improvements were primarily mediated through modulation of host lipid metabolism rather than through reductions in adiposity [28].

### 3.4. Evaluation of Potent Hypoglycemic Activity in C2C12 Cells, Zebrafish, and Mouse Models

#### 3.4.1. Hypoglycemic Effects in Insulin-Resistant C2C12 Cells

**Figure 4:**
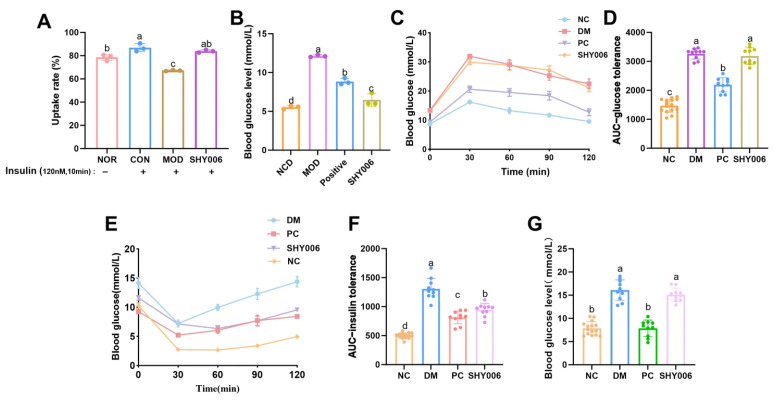
Effects of *L. fermentum* SHY0006 on glucose metabolism in vitro and in vivo. (**A**) Glucose uptake rate (%) in insulin-resistant C2C12 myotubes measured by 2-NBDG assay under insulin stimulation. (**B**) Blood glucose levels (mmol/L) in normal control (NCD), model (MOD), acarbose control (Positive), and SHY0006-treated groups. (**C**) Oral glucose tolerance test (GTT) curves in diabetic mice. (**D**) Area under curve of GTT (AUC-GTT). (**E**) Insulin tolerance test (ITT) curves in diabetic mice. (**F**) Area under curve of ITT (AUC-ITT). (**G**) Fasting blood glucose (FBG, mmol/L) levels in different groups. Data are presented as mean ± SD. Different letters above the bars indicate statistically significant differences among groups (*p* < 0.05).

C2C12 cells have received extensive attention in the research of insulin resistance mechanisms. Skeletal muscle is one of the main tissues for glucose uptake by insulin in the body, and it plays a crucial role in the occurrence and development of diabetes [29]. To determine whether fermentation metabolites from SHY0006 directly regulate glucose uptake, an insulin-resistant (IR) C2C12 model was established and 2-NBDG uptake was quantified (Figure 4A). Under insulin stimulation (120 nM, 10 min), glucose uptake in the control (CON) group increased significantly compared with the normal group (NOR) (*p* < 0.01), confirming preserved insulin responsiveness under physiological conditions. In contrast, the model group (MOD) exhibited a marked reduction in glucose uptake (67.2 ± 0.5%) compared with the CON group (*p* < 0.0001), indicating successful induction of insulin resistance. Importantly, SHY0006 treatment significantly restored glucose uptake to 83.9 ± 1.2%, representing a 24.9% increase relative to the MOD group (*p* < 0.0001). These findings demonstrate that fermentation-derived metabolites from SHY0006 effectively ameliorate insulin resistance and enhance glucose transport capacity in skeletal muscle cells.

#### 3.4.2. Hypoglycemic Effects in Diabetic Zebrafish

The pancreatic structure and function of zebrafish are similar to those of mammals, and the conservation of glucose homeostasis regulation in this animal model makes it suitable for diabetes research [30]. The study further evaluated the hypoglycemic efficacy in a diabetic zebrafish model (Figure 4B). Tissue glucose measurement revealed that the normal control (NC) group exhibited a glucose level of 5.53 ± 0.21 mmol/L, whereas the diabetic model (DM) group showed a significant elevation to 12.13 ± 0.15 mmol/L (*p* < 0.001), confirming successful model establishment. The positive control (PC) group significantly reduced glucose levels to 8.83 ± 0.42 mmol/L compared with the DM group (*p* < 0.01). Notably, SHY0006 treatment further decreased glucose concentration to 6.47 ± 0.81 mmol/L, corresponding to a 46.7% reduction relative to the DM group (*p* < 0.001). The glucose level in the SHY0006 group approached that of the NC group, indicating a pronounced hypoglycemic effect in this vertebrate model.

#### 3.4.3. Hypoglycemic Effects in Diabetic Mice (GTT, ITT, FBG)

The mouse model is a widely employed experimental model in obesity and diabetes research, as it can offer insights into potential therapeutic approaches targeting inflammation, insulin resistance, and other related mechanisms [31]. To further validate the regulatory effect on systemic glucose metabolism, a diabetic mouse model was subjected to glucose tolerance testing (GTT), insulin tolerance testing (ITT), and fasting blood glucose (FBG) measurement (Figure 4C–G). Following glucose challenge, the DM group exhibited significantly higher blood glucose levels (mmol/L) at 30, 60, 90, and 120 min compared with the NC group (*p* < 0.001), demonstrating impaired glucose tolerance (Figure 4C). The area under the curve for glucose (AUC-GTT) was also markedly increased in the DM group (*p* < 0.001 vs. NC) (Figure 4D). SHY0006 administration did not significantly reduce blood glucose levels at individual time points nor the AUC-GTT compared with the DM group (*p* > 0.05), suggesting that this strain may not significantly improve the acute insulin secretory response to glucose challenge, or that its improvement in peripheral insulin sensitivity was insufficient to manifest as an advantage in overall clearance rate under intense glycemic load [32].

In contrast, the ITT revealed a distinct regulatory pattern. After intraperitoneal insulin injection, blood glucose levels (mmol/L) in the DM group remained significantly elevated from 30 to 120 min compared with the NC group (*p* < 0.001), reflecting severe insulin resistance (Figure 4E). SHY0006 treatment significantly reduced blood glucose levels at multiple post-injection time points compared with the DM group (*p* < 0.01). Correspondingly, the AUC-ITT was significantly decreased in the SHY0006 group relative to the DM group (*p* < 0.01), clearly indicating enhanced systemic insulin sensitivity (Figure 4F).

After 30 days of intervention, fasting blood glucose was measured (Figure 4G). The DM group exhibited significantly elevated FBG (16.09 ± 2.21 mmol/L) compared with the NC group (7.87 ± 1.44 mmol/L) (*p* < 0.001). The SHY0006 group showed an FBG level of 15.09 ± 1.45 mmol/L, which was not statistically different from the DM group (*p* > 0.05).

The combined GTT, ITT, and FBG results showed an apparent inconsistency: significant improvement in ITT, but no significant effects on GTT or FBG. This difference may be related to distinct physiological regulatory mechanisms. ITT mainly reflects insulin sensitivity in peripheral tissues such as muscle and adipose tissue; therefore, the observed improvement suggests that SHY0006 may enhance insulin responsiveness and glucose utilization in peripheral tissues [33]. In contrast, GTT and FBG are influenced by multiple factors beyond peripheral insulin sensitivity, including pancreatic β-cell function, hepatic glucose output, and intestinal glucose absorption [34]. Therefore, SHY0006 may contribute to the amelioration of insulin resistance, potentially with a greater effect on peripheral glucose regulation, while its direct impact on hepatic gluconeogenesis or islet secretory function appears limited within the intervention period of this study.

### 3.5. Potential Probiotic Functions Based on Whole-Genome Functional Analysis

**Figure 5:**
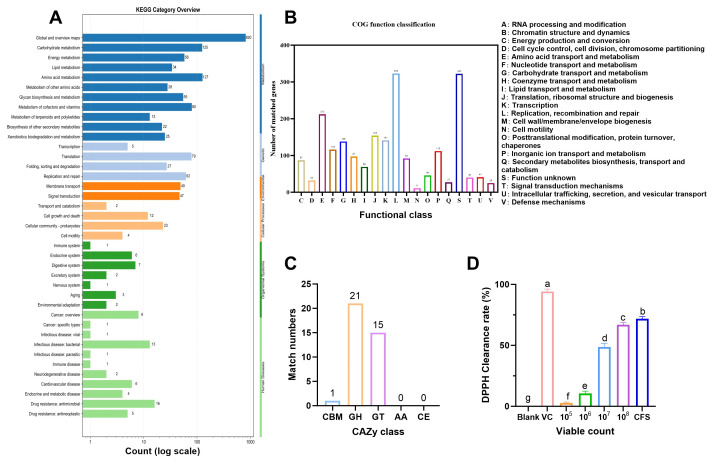
Functional analysis of *L. fermentum* SHY0006. (**A**) Classification map of KEGG metabolic pathways, numbers on the bars indicate the gene count in each annotation; (**B**) COG function classification diagram; (**C**) functional classification of CAZy and the number of corresponding genes; (**D**) DPPH radical scavenging activity of *L. fermentum* SHY0006 at different cell concentrations and of its cell-free supernatant (CFS). Data are presented as mean ± SD. Different letters above the bars indicate statistically significant differences among groups (*p* < 0.05).

The functional genes of *L. fermentum* SHY0006 were comprehensively annotated using multiple databases, including KEGG, COG, and CAZy. KEGG annotation assigned a total of 1775 genes into 6 major categories and 40 functional subclasses, with the highest enrichment observed in carbohydrate metabolism (125 genes), amino acid metabolism (127 genes), and lipid metabolism (34 genes), reflecting SHY0006’s strong metabolic adaptability (Figure 5A). COG annotation further classified 2172 genes into 19 functional categories, predominantly associated with replication and repair (323 genes), carbohydrate transport and metabolism (138 genes), transcription (141 genes), and cell wall/membrane/envelope biogenesis (92 genes), indicating robust genetic stability and versatile metabolic capability (Figure 5B). CAZy analysis identified 20 glycoside hydrolases (GHs), 15 glycosyltransferases (GTs), 1 carbohydrate-binding modules (CBMs), highlighting the strain’s capacity for carbohydrate degradation and polysaccharide synthesis (Figure 5C). Overall, whole-genome functional profiling revealed that SHY0006 possesses a broad repertoire of genes involved in nutrient metabolism, stress tolerance, and probiotic-related functions, supporting its potential as a metabolically versatile and functionally beneficial probiotic candidate.

#### 3.5.1. Antioxidant Capacity

Antioxidant activity contributes to host protection and metabolic regulation [35], prompting further exploration of the strain’s antioxidant capacity. This study evaluated the in vitro antioxidant capacity of SHY0006 through a DPPH radical scavenging assay. The results indicated that the antioxidant activity of the strain was significantly concentration-dependent (Figure 5D). As the viable count increased from 10^5^ to 10^8^ CFU/mL, the DPPH scavenging rate markedly improved from less than 2.8 ± 0.62% to 66.9 ± 1.83%. Most importantly, its cell-free supernatant (CFS) demonstrated the strongest radical scavenging capacity, with a rate of 71.83%, which was comparable to the activity of the full fermentation broth at 10^8^ CFU/mL but significantly lower than that of the positive control, ascorbic acid (VC, 94.17 ± 2.03%).

These findings suggest that both SHY0006 cells and their fermentation supernatant possess antioxidant potential. The strong activity observed in the cell-free supernatant (CFS) indicates that extracellularly released metabolites may contribute substantially to this effect. However, the specific antioxidant compounds were not identified in the present study. These results support the potential of SHY0006 to mitigate oxidative stress in the gut microenvironment.

#### 3.5.2. Genomic Basis of Vitamin (B2, B9) Biosynthesis

The complete riboflavin (vitamin B_2_) biosynthesis pathway is considered an important probiotic-associated trait, as riboflavin not only serves as an essential micronutrient but also participates in antioxidant defense and cellular energy metabolism [36]. Folate (vitamin B_9_), in its biologically active tetrahydrofolate (THF) form, plays a central role in one-carbon metabolism, nucleotide biosynthesis, and methylation reactions. Genome annotation and KEGG pathway reconstruction revealed that *L. fermentum* SHY0006 possesses a complete de novo vitamin B_2_ and B_9_ biosynthesis pathway.

**Figure 6:**
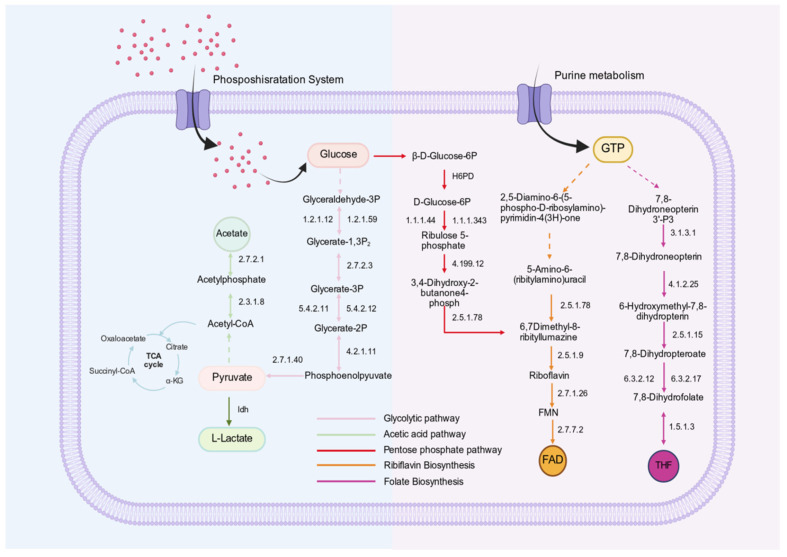
The main pathways and metabolites of *L. fermentum* SHY0006 in glucose absorption and utilization, fatty acid biosynthesis, and vitamin B biosynthesis. The dotted arrows indicate the omitted parts in the pathways.

Genome annotation and KEGG pathway reconstruction revealed that *L fermentum* SHY0006 harbors complete de novo biosynthetic pathways for both riboflavin and folate, as shown in Figure 6. The riboflavin pathway initiates from GTP and ribulose-5-phosphate precursors and proceeds through the coordinated action of ribA/ribD, ribB, ribH, and ribE, ultimately generating riboflavin, which can be further converted into its active cofactor forms FMN and FAD. In parallel, the folate biosynthesis pathway begins with GTP-derived dihydroneopterin intermediates and involves key enzymes including folK, folP, folC, and folA, leading to the formation of tetrahydrofolate (THF).

The presence of all essential enzymatic components required for these uninterrupted cascades indicates that SHY0006 possesses fully functional riboflavin and folate biosynthetic pathways, highlighting its potential to contribute to host vitamin supply and metabolic regulation.

#### 3.5.3. Carbohydrate Metabolism and Short-Chain Fatty Acid (SCFA) Production

Carbohydrate utilization is essential for probiotic survival, colonization, and functional activity in the gastrointestinal tract [37]. Similar to other LAB, SHY0006 relies on the phosphotransferase system (PTS) to transport and phosphorylate a variety of fermentable carbohydrates [38]. Genome annotation revealed a broad repertoire of PTS-associated EIIA/B/C components targeting multiple substrates, including glucose/maltose/α-glucoside/β-glucoside (Crr), sucrose (ScrA), mannose (ManX/Y/Z), cellobiose (CelB), galactitol (GatC), sorbitol (SrlB, SrlE), and trehalose (TreP/Crr), indicating strong metabolic flexibility and adaptability to diverse carbon sources.

KEGG pathway reconstruction further demonstrated that these imported carbohydrates can be efficiently channeled into a complete glycolytic pathway (module M00002), enabling the rapid conversion of glucose-6-phosphate and other phosphorylated sugars into pyruvate, as shown in Figure 6. Once formed, pyruvate is metabolized through two major fermentation routes in SHY0006. First, a functional lactate dehydrogenase (*ldh*) gene mediates the homolactic fermentation pathway, reducing pyruvate to lactate. Second, SHY0006 also possesses a complete acetate-producing branch, with both phosphotransacetylase (Pta, EC 2.3.1.8) and acetate kinase (AckA, EC 2.7.2.1) identified in the genome. Together, these enzymes constitute the canonical Pta-AckA pathway, in which pyruvate-derived acetyl-CoA is converted into acetyl-phosphate by Pta and subsequently dephosphorylated by AckA to yield acetate, generating an additional molecule of ATP through substrate-level phosphorylation. Lactate contributes to gut acidification and host glucose regulation [39], whereas acetate is a key microbial metabolite associated with modulation of lipid metabolism, AMPK activation, appetite regulation, and improvement in insulin sensitivity [40].

Collectively, SHY0006 demonstrates a favorable safety profile and extracellular antioxidant activity, comprehensive carbohydrate metabolism and SCFA biosynthetic potential, and reproducible triglyceride-lowering and insulin-sensitizing effects across multiple models. These integrated genomic and phenotypic findings support SHY0006 as a promising functional probiotic candidate for modulation of host lipid and glucose metabolism.

### 3.6. Environmental Stress Tolerance

To assess the environmental adaptability of *L. fermentum* SHY0006, genome annotation was conducted to identify stress-related genes (Table S1). Genes associated with temperature tolerance (dnaK, dnaJ, clpB, cspA-cspC, cshB), osmotic stress (opuA, opuC, grpE, osmV), acid stress (atpA–atpH), bile salt resistance (luxS, oppA, argS, clp), and Additionally, several general stress response genes (uspA, usp2) were present. The presence of these genes indicates that SHY0006 possesses the genetic potential to withstand diverse environmental challenges, supporting its survival and functionality under gastrointestinal and food-related stress conditions. Beyond environmental resilience, the ability to adhere to gut surfaces and interact with gut microbiota plays a crucial role in colonization and host modulation [41].

#### 3.6.1. Tolerance to Different Temperatures, pH and NaCl

**Figure 7:**
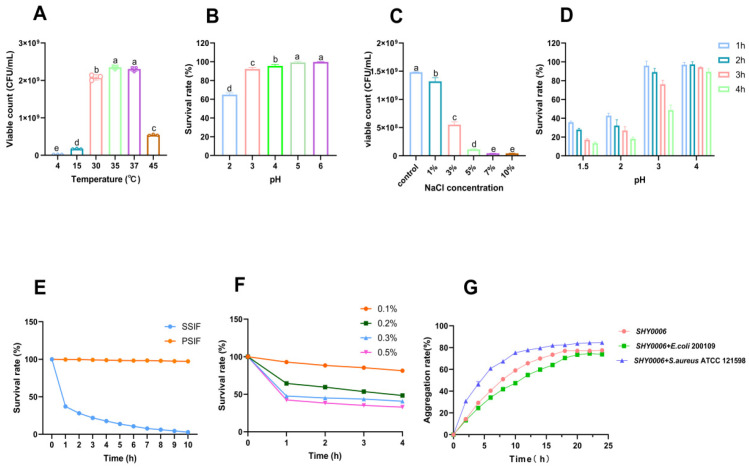
Environmental tolerance and aggregation ability of *Limosilactobacillus fermentum* SHY0006. (**A**) Viable counts (CFU/mL) of *L. fermentum* SHY0006 at different temperatures; (**B**) Survival rates (%) of *L. fermentum* SHY0006 in different pH; (**C**) viable counts (CFU/mL) of *L. fermentum* SHY0006 in different concentrations of NaCl; (**D**) survival rate (%) of *L. fermentum* SHY0006 in simulated gastric fluid (SGF) at different pH values (pH 1.5–4.0) over time (1–4 h); (**E**) survival rate (%) of *L. fermentum* SHY0006 in simulated intestinal fluids (PSIF and SSIF) over time. SGF: simulated gastric fluid; PSIF: primary simulated intestinal fluid; SSIF: secondary simulated intestinal fluid; (**F**) survival rates (%) of *L. fermentum* SHY0006 in different concentrations of bile salt. (**G**) Self-aggregation rate (%) of *L. fermentum* SHY0006 and its co-aggregation rate (%) with other tested bacteria. Data are expressed as mean ± SD from at least three independent experiments. Statistical analysis was performed using one-way ANOVA followed by post hoc comparison. Different lowercase letters denote significant differences (*p* < 0.05).

In the temperature tolerance assay (Figure 7A), SHY0006 exhibited optimal growth at 35 °C (2.35 ± 0.06 × 10^9^ CFU/mL), followed closely by 37 °C (2.30 ± 0.06 × 10^9^ CFU/mL). Growth declined substantially at 4 °C, 25 °C, and 45 °C, indicating that SHY0006 is a mesophilic strain with a narrow optimal temperature window, consistent with typical physiological characteristics of Lactobacillus species, aligning with findings for *L. fermentum* 664 [42].

The acid tolerance of SHY0006 was further evaluated by incubating the strain in physiological saline adjusted to pH 2–6 for 4 h (Figure 7B). Survival increased markedly with rising pH. SHY0006 retained 64.85% ± 1.89% viability at pH 2, demonstrating moderate resistance to highly acidic conditions. Survival improved significantly at pH 3–4 (92.26% ± 1.56% and 95.57% ± 1.51%, respectively), and nearly complete survival (>99%) was observed at pH 5 and 6, Similarly, *L. fermentum* MB1 [43] also demonstrates strong pH tolerance, indicating good resilience to gastric acidity—particularly under postprandial conditions (pH ≥ 3).

NaCl tolerance testing revealed robust viability at low salinity (≤1% NaCl; 1.32 × 10^9^ CFU/mL, 89.2% of control) but a pronounced viability decline at ≥3% NaCl, with counts falling below 1 × 10^8^ CFU/mL at ≥5% NaCl (Figure 7C). These results indicate that SHY0006 displays moderate salt tolerance, sustaining relatively good growth under low-salinity conditions (≤1%), whereas high osmotic pressure (>3% NaCl) markedly compromises its viability.

#### 3.6.2. Tolerance to Simulated Gastroenteric Fluid

The gastrointestinal environment of the human body is unfavorable for the survival of probiotics and may even lead to their death. Therefore, the tolerance of probiotics to gastrointestinal fluids is a prerequisite for the colonization of strains with probiotic potential [44]. pH values of 1.5, 2.0, 3.0 and 4.0 were selected to assess gastric fluid tolerance (Figure 7D). SHY0006 demonstrated exceptional stability in SGF at pH 4.0, maintaining a survival rate as high as 89.47 ± 3.21% even after 4 h of exposure. Under pH 3.0 conditions (representing postprandial gastric environment), the strain retained a survival rate of 48.70 ± 5.39% after 4 h. In contrast, under strongly acidic conditions at pH 1.5 and pH 2.0, although the strain’s viability decreased, it still achieved survival rates of 13.46 ± 1.16% and 18.11 ± 1.86% after 4 h, respectively. This sharp decline likely stems from acid-induced damage to cell membranes and inhibition of key enzyme activities [45]. Given that the gastric pH in humans typically rises above 3.0 after meals, the strong tolerance demonstrated by SHY0006 under pH 3.0 and 4.0 conditions indicates that the viable bacterial count remaining after 4 h of treatment at pH 3.0 is expected to be sufficient to reach the concentration required for intestinal colonization and probiotic function [46]. This establishes a solid foundation for its application as a probiotic.

#### 3.6.3. Tolerance to Simulated Intestinal Conditions

The tolerance of LAB to simulated intestinal conditions and bile salts is a critical determinant of their probiotic efficacy, as it directly influences their survival and functionality in the host gastrointestinal tract (GIT) [47,48]. The strain SHY0006 exhibited outstanding stability in the primary simulated intestinal fluid (PSIF, pH 8.0), maintaining a survival rate above 97% even after 10 h of incubation. This indicates a high inherent resistance to pancreatic enzymes such as trypsin. In the secondary simulated intestinal fluid (SSIF, pH 8.0, containing 0.3% bile salts), the survival rate decreased sharply over time to 17.59 ± 0.88% after 4 h (Figure 7E). A more detailed analysis in MRS broth supplemented with varying bile salt concentrations confirmed a clear dose-dependent inhibitory effect (Figure 7F). After 4 h of exposure—a critical timeframe for intestinal transit—the survival rates were 92.89 ± 1.60%, 64.45 ± 2.44%, 47.59 ± 0.73%, and 42.42 ± 0.78% at concentrations of 0.1%, 0.2%, 0.3%, and 0.5% bile salts, respectively. The detrimental effect of bile salts, known to disrupt the bacterial cell membrane and induce oxidative stress, is the primary reason for this observed reduction [49]. These findings suggest that SHY0006 possesses moderate bile salt tolerance and can survive transient exposure to gastrointestinal conditions, supporting its potential as a probiotic candidate.

#### 3.6.4. Adhesion-Related Properties

The self-aggregation and co-aggregation abilities of LAB are critical mechanisms that influence their probiotic efficacy, microbial interactions, these properties enable LAB to form multicellular clusters (self-aggregation) or bind with other microbial species (co-aggregation), which can enhance their colonization, pathogen exclusion, and functional stability in complex ecosystems [50,51]. The in vitro assessment of probiotic properties demonstrated that the strain SHY0006 possesses excellent self-aggregation and co-aggregation capabilities (Figure 7G). The self-aggregation capacity increased rapidly over time, reaching a high final value of over 77% after 24 h, indicating a strong potential for biofilm formation and gut colonization. Notably, SHY0006 exhibited a superior co-aggregation ability with pathogenic bacteria, particularly against *Staphylococcus aureus*, with a rate exceeding 85%. In comparison, the co-aggregation rate with *E. coli* reached approximately 74% after 24 h, which was lower than that observed for *S. aureus.* This difference may be related to differences in surface structure and membrane composition between Gram-negative and Gram-positive bacteria, which can influence intercellular adhesion efficiency. This strong co-aggregation suggests that SHY0006 could potentially inhibit the colonization and proliferation of pathogens in the gastrointestinal tract through physical exclusion and competition for adhesion sites [52]. The aggregation characteristics of SHY0006, therefore, contribute significantly to its potential probiotic function in maintaining intestinal microbial homeostasis and defending against infections.

### 3.7. Study Limitations and Future Perspectives

Despite the promising findings, several limitations should be considered. The molecular mechanisms underlying the metabolic effects of SHY0006 were not directly investigated in the present study. Although improvements in lipid metabolism and insulin sensitivity were observed, the specific signaling pathways and regulatory genes involved remain unclear and require further investigation.

In addition, the bioactive metabolites responsible for the lipid-lowering effects were not identified. Probiotic fermentation products, including organic acids, short-chain fatty acids, and other microbial metabolites, are known to influence host metabolic regulation [53]. Integrating metabolomic and transcriptomic analyses in future studies would help identify the functional metabolites and clarify the molecular mechanisms underlying the observed metabolic benefits. The present work also mainly focused on metabolic phenotypes. Further studies examining the modulation of gut microbiota composition and host metabolic signaling pathways may provide deeper insight into the probiotic mechanisms of SHY0006. A more comprehensive investigation of these aspects will contribute to a better understanding of the functional properties of this strain and support its potential application in metabolic health management.

### 3.8. Traditional Fermented Foods as Sources of Novel Functional Probiotics

Traditional fermented foods represent an important reservoir of diverse microorganisms with potential probiotic properties. In recent years, increasing attention has been given to indigenous fermented foods as valuable sources for isolating novel functional strains with health-promoting potential [54]. In this study, SHY0006 was isolated from traditional Miao sour soup, a characteristic fermented food produced by ethnic communities in Southwest China. The discovery of a metabolically beneficial strain from this traditional food highlights the significant microbial diversity present in ethnic fermented foods and underscores their importance as sources of probiotic candidates. The identification and functional characterization of such strains not only contribute to the scientific understanding of traditional fermented foods but also provide opportunities for developing new probiotic ingredients and functional food products targeting metabolic health.

## 4. Conclusions

This study systematically characterized *Limosilactobacillus fermentum* SHY0006 as a safe and metabolically functional probiotic candidate. Whole-genome sequencing confirmed the absence of virulence-associated determinants and acquired antibiotic resistance genes, supporting its genomic safety. Functionally, SHY0006 demonstrated significant regulatory effects on lipid and glucose metabolism in both cells and animal models. In hyperlipidemic mice, the strain significantly reduced hepatic triglyceride accumulation and favorably modulated serum lipid parameters, including LDL-C, HDL-C, and free fatty acids. In diabetic mice, SHY0006 markedly improved insulin tolerance test (ITT) performance, indicating enhanced systemic insulin sensitivity and alleviation of insulin resistance. Genomic analysis further revealed complete biosynthetic pathways for riboflavin and folate, together with extensive carbohydrate utilization capacity, highlighting its metabolic versatility. In addition, the strain exhibited strong environmental adaptability and stress tolerance, supporting its potential viability in food matrices and gastrointestinal conditions. Collectively, the integrated genomic and experimental evidence suggests that SHY0006 represents a promising probiotic candidate for functional food applications aimed at improving metabolic health.

## Figures and Tables

**Table 1:** Nutritional composition of the high-fat diet (HFD) and control diet (NCD) used in the animal experiment.

Nutritional Component	High-Fat Diet		Control Diet	
	Mass Ratio (%)	Energy Ratio (%)	Mass Ratio (%)	Energy Ratio (%)
Protein	26.2	20	19.2	20
Carbohydrates	26.3	20	67.3	70
Fat	34.9	60	4.3	10

**Table 2:** Analysis of antibiotic resistance.

Classification	Antibiotics	Inhibition Zone (mm)	Antibiotic Resistance
β-lactams	Penicillin	29.53 ± 1.04	S
β-lactams	Carbenicillin	30.79 ± 0.75	S
β-lactams	Piperacillin	29.38 ± 1.07	S
β-lactams	Ampicillin	27.91 ± 1.48	S
β-lactams	Cefazolin	29.01 ± 0.8	S
β-lactams	Ceftriaxone	25.57 ± 1.15	S
β-lactams	Cefoperazone	18.35 ± 0.88	I
β-lactams	Cephalexin	24.11 ± 1.05	S
β-lactams	Cefradine	23.83 ± 0.39	S
β-lactams	Cefuroxime	20.53 ± 1.22	S
β-lactams	Ceftazidime	17.63 ± 0.5	I
Macrolides	Erythromycin	30.04 ± 0.73	S
Macrolides	Midecamycin	30.13 ± 0.93	S
Quinolones	Ofloxacin	13.98 ± 0.21	I
Aminoglycosides	Amikacin	15.25 ± 0.32	I
Aminoglycosides	Gentamicin	15.37 ± 0.39	S
Aminoglycosides	Kanamycin	12.09 ± 0.65	R
Aminoglycosides	Neomycin	16.81 ± 1.07	S
Tetracyclines	Tetracycline	22.81 ± 0.22	S
Tetracyclines	Doxycycline	25.79 ± 0.81	S
Tetracyclines	Minocycline	23.27 ± 0.82	S
Sulfonamides	Compound Sulfamethoxazole	17.38 ± 0.67	S
Amphenicols	Chloramphenicol	29.07 ± 1.09	S
Lincosamides	Clindamycin	31.09 ± 0.69	S
Glycopeptides	Vancomycin	0 ± 0	R
Nitrofurantoin	Furazolidone	22.42 ± 0.83	S

Abbreviations: S, susceptible; I, intermediate; R, resistant.

## Data Availability

The original contributions presented in this study are included in the article/Supplementary Material. Further inquiries can be directed to the corresponding authors.

## Supplementary Materials

The following supporting information can be downloaded at: https://www.mdpi.com/article/10.3390/foods15091508/s1.

## Abbreviations

The following abbreviations are used in this manuscript:

LAB	lactic acid bacteria
WGS	whole-genome sequencing
SCFA	short-chain fatty acid
TG	triglyceride
TC	total cholesterol
LDL-C	low-density lipoprotein cholesterol
NCD	normal chow diet
GTT	glucose tolerance test
ITT	insulin tolerance test
FBG	fasting blood glucose
IR	insulin resistance
ORO	Oil Red O
PTS	phosphotransferase system
