# Supplementary material for: Whole-Genome-Guided Functional Characterization of Limosilactobacillus fermentum SHY0006 Reveals Hypolipidemic Activity and Improvement in Insulin Resistance
Source: Foods. 2026 Apr 27;15(9):1508. doi: 10.3390/foods15091508 (PMC13163674; doi:10.3390/foods15091508)
Supplement: Supplementary file 1 [file foods-15-01508-s001.zip › foods-4229058-supplementary.pdf]

Table S1. Probiotic characteristic genes

Function	gene	Description	SHY0006 locus
Temperature tolerance	*xylA*	xylose isomerase	ACWNPI_00990
	cspA	cold shock protein	ACWNPI_00412
	cspB	cold shock protein	ACWNPI_00412
	cspC	cold shock protein	ACWNPI_00412
	dnaJ	molecular chaperone DnaJ	ACWNPI_02040
	dnaK	molecular chaperone DnaK	ACWNPI_02041
	hrcA	heat-inducible transcriptional repressor	ACWNPI_02043
	clpC	ATP-dependent Clp protease ATP-binding subunit ClpC	ACWNPI_02009
	groEL	chaperonin GroEL	ACWNPI_02174
	groES	chaperonin GroES	ACWNPI_02173
Oxidative Stress	*lexA*	repressor LexA	ACWNPI_00752
	recA	recombination protein RecA	ACWNPI_00098
	trxA	thioredoxin	ACWNPI_00115
	trxB	thioredoxin reductase (NADPH)	ACWNPI_00782
	feoB	ferrous iron transport protein B	ACWNPI_00935
	cydA	cytochrome bd ubiquinol oxidase subunit	ACWNPI_00588
	cydC	ATP-binding cassette, subfamily C	ACWNPI_00591
	ndh	NADH:quinone reductase (non-electrogenic)	ACWNPI_01963
	msrA	peptide-methionine (S)-S-oxide reductase	ACWNPI_01576
	msrB	peptide-methionine (R)-S-oxide reductase	ACWNPI_00914
	msrC	L-methionine (R)-S-oxide reductase	ACWNPI_00917
	hslO	molecular chaperone Hsp33	ACWNPI_00131
	tpx	thioredoxin-dependent peroxiredoxin	ACWNPI_00083
	mutT	MTH2; 8-oxo-dGTP diphosphatase	ACWNPI_00730
	msrB	peptide-methionine (R)-S-oxide reductase	ACWNPI_00914
	ahpC	NADH-dependent peroxiredoxin subunit C	ACWNPI_00995
osmotic shock tolerance	*opuA*	osmoprotectant transport system ATP-binding protein	ACWNPI_00220
	opuC	osmoprotectant transport system substrate-binding protein	ACWNPI_00222
	opuCA	osmoprotectant transport system ATP-binding protein	ACWNPI_00220

	opuCB	osmoprotectant transport system permease protein	ACWNPI_00219
	opuCC	osmoprotectant transport system substrate-binding protein	ACWNPI_00222
	grpE	molecular chaperone GrpE	ACWNPI_02042
	opuCB	osmoprotectant transport system permease protein	ACWNPI_00219
	osmV	osmoprotectant transport system ATP-binding protein	ACWNPI_00220
	atpC	F-type H+-transporting ATPase subunit epsilon	ACWNPI_01533
	atpD	F-type H+/Na+-transporting ATPase subunit beta	ACWNPI_01532
	atpG	F-type H+-transporting ATPase subunit gamma	ACWNPI_01531
	atpH	F-type H+-transporting ATPase subunit delta	ACWNPI_01529
	atpF	F-type H+-transporting ATPase subunit b	ACWNPI_01528
	atpB	F-type H+-transporting ATPase subunit a	ACWNPI_01526
	atpE	F-type H+-transporting ATPase subunit c	ACWNPI_01527
	recA	recombination protein RecA	ACWNPI_00098
	aspS	aspartyl-tRNA synthetase	ACWNPI_00840
acid stress	*cspA*	cold shock protein	ACWNPI_00412
	gpmA	2,3-bisphosphoglycerate-dependent phosphoglycerate mutase	ACWNPI_00016
	glmU	UDP-N-acetylglucosamine pyrophosphorylase	ACWNPI_01321
	glmS	glutamine---fructose-6-phosphate transaminase (isomerizing)	ACWNPI_00969
	glmM1	Phosphoglucosamine mutase	ACWNPI_00968
	glmM2	Phosphoglucosamine mutase	ACWNPI_00968
	yphJ	4-carboxymuconolactone decarboxylase	ACWNPI_00500
	grpE	molecular chaperone GrpE	ACWNPI_02042
	atpA	F-type H+/Na+-transporting ATPase subunit alpha	ACWNPI_01530
	clpE	ATP-dependent Clp protease ATP-binding subunit ClpE	ACWNPI_01900
	clpP_1	ATP-dependent Clp protease proteolytic subunit precursor	ACWNPI_01269

	clpP_2/clpP	ATP-dependent Clp protease proteolytic subunit precursor	ACWNPI_01269
	copA	P-type Cu+ transporter	ACWNPI_00244
	dltA	D-alanine--poly(phosphoribitol) ligase subunit 1	ACWNPI_01180
	dltD	D-alanine transfer protein	ACWNPI_01177
	dnaJ	molecular chaperone DnaJ	ACWNPI_02040
	dnaK	molecular chaperone DnaK	ACWNPI_02041
	eno	enolase 1/2/3	ACWNPI_00953
	eno2	enolase 1/2/3	ACWNPI_00953
	gapA	glyceraldehyde 3-phosphate dehydrogenase (phosphorylating)	ACWNPI_00950
	groL/groEL	chaperonin GroEL	ACWNPI_02174
	groS/groES	chaperonin GroES	ACWNPI_02173
	grpE	molecular chaperone GrpE	ACWNPI_02042
	guaA	GMP synthase (glutamine-hydrolysing)	ACWNPI_01978
	ldh_1/ldh	L-lactate dehydrogenase	ACWNPI_01097
	pgk	phosphoglycerate kinase	ACWNPI_00951
	pyk	pyruvate kinase	ACWNPI_00874
	recA	recombination protein RecA	ACWNPI_00098
	tpiA	triosephosphate isomerase	ACWNPI_00952
	uvrA/uvrA_1	excinuclease ABC subunit A	ACWNPI_01355
	yjbM	GTP pyrophosphokinase	ACWNPI_01662
	ywaC	GTP pyrophosphokinase	ACWNPI_01662
	argS	arginyl-tRNA synthetase	ACWNPI_00370
	clp	ATP-dependent Clp protease	ACWNPI_01269
	dps	starvation-inducible DNA-binding protein	ACWNPI_01023
	glf	UDP-galactopyranose mutase	ACWNPI_01619
	glnA	glutamine synthetase	ACWNPI_00310
	luxS	S-ribosylhomocysteine lyase	ACWNPI_01468
	pdhD_2/lpdA	dihydrolipoyl dehydrogenase	ACWNPI_00539
Bile resistance	*pepO*	putative endopeptidase	ACWNPI_01801
	ponA/pbp1A	penicillin-binding protein 1A	ACWNPI_02111
	pyrG	CTP synthase	ACWNPI_01314
	rplD	large subunit ribosomal protein L4	ACWNPI_01257
	rplE	large subunit ribosomal protein L5	ACWNPI_01246
	rplF	large subunit ribosomal protein L6	ACWNPI_01243
	rpsC	small subunit ribosomal protein S3	ACWNPI_01252
	rpsE	small subunit ribosomal protein S5	ACWNPI_01241
	srtA	sortase A	ACWNPI_00149

Table S2. CARD (RGI) result

Analysis item	Description
Database	Comprehensive Antibiotic Resistance Database (CARD)
Tool	Resistance Gene Identifier (RGI)
Screening criteria	Strict and perfect hits
Result	No acquired antibiotic resistance genes detected
